# Linking child travel routes and routine health data

**DOI:** 10.23889/ijpds.v1i1.302

**Published:** 2017-04-19

**Authors:** Amy Mizen, Sarah Rodgers, Richard Fry, Ronan Lyons

**Affiliations:** Swansea University

## Objectives

Linking routinely collected health and environment data can allow for large scale evaluations of how our environment impacts our health. Our data linkage approach advances previous research where residence-based environmental exposures were anonymously linked in the SAIL databank using Residential Anonymous Linking Fields (RALFs). The dose-response relationship between exposure to food and dietary intake has not been widely investigated. Previous research found conflicting views on whether increased environmental exposure to unhealthy food contributes to higher BMIs. This may have been due to different methodological approaches, including imprecise exposures, small numbers, and the use of self-reported BMIs.

## Approach

This investigation calculated food exposure environments for routes from all homes to and from school. A Geographic Information System was used to calculate the environmental exposures along all potential routes up to a maximum age-appropriate walking distance from each school. Once within the SAIL databank we selected relevant routes using linked demographic and pupil datasets. To maintain privacy, the primary (doctoral) researcher generating the environmental exposures, did not have access to the final household-level exposure data in their identifiable form. The researcher automated their method so a second researcher could run the GIS analysis. Accuracy of modelled exposures will be compared with actual routes collected from GPS traces of children walking to school.

## Results

Removing access to the final identifiable household-level route exposures enabled the primary researcher to complete analysis on the combined household and individual-level data within the secure environment. The environmental exposures were linked with routine health data from the SAIL databank; including BMI as an indicator of obesity. BMI data for 4-5 year olds, and a sample of 1300 13-14 year olds were linked to associated environmental exposures.

## Conclusion

Depending on modelled accuracy, a GIS and data linkage approach may allow the investigation of natural experiments and intervention evaluation at the scale of the total population. This is the first step towards anonymously modelling part of the daily exposure environment using routine data. A limitation is the lack of routinely collected BMI data for older children and teenagers an age when they are more likely to have the option to choose to buy food on the school route. This work will have many potential applications, including the delivery and evaluation of multiple school and workplace commuting interventions.

